# Eye Pain Caused by Epithelial Damage in the Central Cornea in Aqueous-Deficient Dry Eye

**DOI:** 10.3390/diagnostics14010030

**Published:** 2023-12-22

**Authors:** Yamato Yoshikawa, Norihiko Yokoi, Natsuki Kusada, Hiroaki Kato, Rieko Sakai, Aoi Komuro, Yukiko Sonomura, Chie Sotozono

**Affiliations:** 1 Department of Ophthalmology, Osaka Medical and Pharmaceutical University, Takatsuki-City 569-8686, Japan; yamt.0dec0@gmail.com; 2 Department of Ophthalmology, Kyoto Prefectural University of Medicine, Kyoto 602-0841, Japan; nkusada9@koto.kpu-m.ac.jp (N.K.); hiro-kat@koto.kpu-m.ac.jp (H.K.); r-sakai@menicon.co.jp (R.S.); akomuro@koto.kpu-m.ac.jp (A.K.); yukky@ymail.plala.or.jp (Y.S.); csotozon@koto.kpu-m.ac.jp (C.S.)

**Keywords:** dry eye, aqueous-deficient dry eye, eye pain, pain quantitative measuring device, questionnaire

## Abstract

In this study, the severity of eye pain (EP) and associated objective findings were evaluated in aqueous-deficient dry eye (ADDE) patients using PainVision^®^, a quantitative pain-measuring device. This study involved 53 eyes of 53 ADDE patients (6 males and 47 females; mean age: 64.4 ± 13.4 [mean ± SD] years). Of those, 18 eyes of 18 patients underwent punctal occlusion, and EP and objective findings in those patients were evaluated before and after treatment. In all patients, the severity of EP as measured by PainVision^®^ was assessed using the Pain Degree (PD). The median PD for the 53 patients was 30.6 µA/µA (interquartile range, 16.9–93.2), and the nasal and central corneal staining score and the upper lid-wiper epitheliopathy score were significantly correlated with PD (R = 0.33, 0.33, and 0.28, respectively) (all: *p* < 0.05). Using the least squares method, the central corneal staining score most significantly affected PD. In the 18 cases that underwent punctal occlusion, PD was significantly reduced (median PD: 24.8 to 7.1 µA/µA; *p* < 0.0001). Using the least squares method, the central corneal staining score and tear meniscus radius were significantly more influential as factors contributing to PD before and after treatment, and central corneal epithelial damage was the factor most associated with ADDE-related EP.

## 1. Introduction

According to the official Asia Dry Eye Society (ADES) definition, dry eye (DE) is defined as “a multifactorial disease characterized by unstable tear film causing a variety of symptoms and/or visual impairment, potentially accompanied by ocular surface damage” [1], and, as reported in a study by the Tear Film & Ocular Surface Society [2], the prevalence of DE is quite high, as from 5 to 30% of the world population is affected by the disease.

DE has many symptoms, of which eye pain (EP) is a serious clinical problem. As findings have shown in several previous studies, DE symptoms are not directly associated with the signs of DE [3,4,5]. In other words, various symptoms are caused by DE that are difficult to treat because they often do not correlate with the objective findings of DE. Hence, we investigated the relationship between DE and EP in an attempt to clarify the objective findings associated with EP and elucidate the specific targets requiring treatment.

Several previous studies have investigated the association between DE and EP [6,7,8,9], and although neuropathic ocular pain is a very important EP-associated disease concept, it remains controversial as to whether or not it directly indicates DE [10,11]. Moreover, although patient questionnaires have generally been used in most previous reports to assess EP, assessment via that method is susceptible to case-specific psychological and personality factors [12]. Recently, PainVision^®^ (PS-2100; Nipro Corporation, Osaka, Japan), a new painless electrical stimulation system for the quantitative assessment of pain intensity via substituting pain with a different sensory stimulation, has been developed and is currently mainly being used in the field of anesthesiology and in pain clinics in Japan [13,14,15,16,17,18,19]. In a recent study [20], we used PainVision^®^ to evaluate EP in DE cases, and we found that the system allowed for easy measurement and assessment of pain and an analysis of its correlation with objective findings. Moreover, we found that the objective finding most significantly associated with EP was the classification of DE subtypes [20]. This finding revealed that without classifying DE into subtypes, it is difficult to link EP to other objective findings. This classification of DE is based on the fluorescein breakup pattern (FBUP) in the following three subtypes: (1) aqueous-deficient DE (ADDE), (2) decreased wettability DE (DWDE), and (3) increased evaporation DE (IEDE) [21,22]. These three DE subtypes have completely different ocular objective findings, and even within each subtype the severity of EP varies. Thus, it important to clearly elucidate the association between objective findings and EP severity in each of these three subtypes.

The purpose of this present study was to investigate the relationship between the objective findings of corneal epithelial damage and EP severity in ADDE patients using the PainVision^®^ device, and then evaluate the pain and assess the findings in each patient before and after undergoing punctal occlusion treatment.

## 2. Materials and Methods

The protocols of this study were approved by the Ethics Committee and the Institutional Review Board of Kyoto Prefectural University of Medicine, Kyoto, Japan (approval no. ERB-C-1233-3), and were carried out in accordance with the tenets set forth in the Declaration of Helsinki. Prior written informed consent was obtained from all subjects after a detailed explanation of the nature of the study and possible consequences associated with participation.

### 2.1. Subjects

This study involved 53 eyes of 53 ADDE patients with EP seen at the Dry Eye Outpatient Clinic at the Kyoto Prefectural University of Medicine Hospital. In the DE patients seen, FBUP was classified at initial presentation, and the cases in which the FBUP was ‘line break’ (*n* = 26 cases) or ‘area break’ (*n* = 27 cases) were included in this study as ADDE. Of those 53 ADDE cases, 18 eyes of 18 patients were treated with punctal occlusion, i.e., a treatment used for severe ADDE with area break and for cases that did not respond to topical medications such as diquafosol sodium ophthalmic suspension, rebamipide ophthalmic suspension, and steroid eye drops. When both eyes were indicated for punctal occlusion treatment, the data of the eye treated first were selected. Exclusion criteria included any subjects who were using glaucoma eye drops, who had cardiac pacemakers or an equivalent, who were pregnant or had planned pregnancy during the study period, who were within a 3-month range post ophthalmic surgery (including eyelid surgery, glaucoma surgery, and surgery for ocular surface disease), and who were contact lens users. Moreover, cases with severe conjunctivochalasis (and those in which examination of the tear meniscus could not be performed), symblepharon, trichiasis, and deformities of the eyelids and ocular surface were excluded, as those conditions can cause EP that is different from DE-associated EP. Ocular surface findings and EP assessed via both questionnaires and PainVision^®^ were evaluated, and in the 18 patients who underwent punctal occlusion treatment, EP findings before treatment and postoperatively after 1 month or more were compared.

### 2.2. DE-Related Symptoms Evaluated Using DE-System Questionnaire Visual Analogue Scale (DSQ-VAS)

The ‘Visual Analogue Scale’ (VAS) is a tool used for the evaluation of subjective symptoms that cannot be directly measured, and, via this method, the DE-related symptom is evaluated by answering “0” for no symptoms and “100” for the most severe symptoms.

In this study, prior to the ocular surface examinations, the VAS was used to evaluate the DE-related symptoms in a questionnaire, i.e., EP, dryness, blurred vision, sensitivity to light, eye fatigue, heavy eyelids, foreign body sensation, difficulty in opening the eye, redness, tearing, itchiness, and discharge. This questionnaire, termed ‘DQS-VAS’, was used in our previous study [20].

### 2.3. DE-Related EP Symptoms Evaluated Using Short-Form McGill Pain Questionnaire 2 (SF-MPQ-2)

In this study, the SF-MPQ-2, a questionnaire that evaluates the pain experienced by each of the 22 pain descriptors using an 11-point numerical rating scale (0 = “none” to 10 = “worst possible”), was used to evaluate the severity of EP [23]. Although this questionnaire was also used in our previous study, it is primarily used in the field of anesthesiology and at specialized pain clinics, and is composed of the following four pain subscales: (1) continuous pain (throbbing pain, cramping pain, gnawing pain, aching pain, heavy pain, and tenderness), (2) intermittent pain (shooting pain, stabbing pain, sharp pain, splitting pain, electric-shock pain, and piercing), (3) neuropathic pain (hot-burning pain, cold-freezing pain, pain caused by light touch, itching, tingling or ‘pins and needles’, and numbness), and (4) affective descriptors (tiring-exhausting, sickening, fearful, and punishing-cruel).

A total pain point was then computed by the ratings provided by the subjects across all questions, while the pain subscale points were derived from ratings to questions that comprised the respective scales.

### 2.4. Quantitative Evaluation of EP Using PainVision^®^

In this study, PainVision^®^ was used to objectively evaluate the severity of EP under natural blinks. As previously reported [13,14], the electrode part of the device that transmits electrical current is attached to the medial forearm. Briefly, the current perception threshold (CPT), defined by the minimum electrical stimulation that could be sensed by the subject, was measured. Next, the pain equivalent current (PEC), defined by the electrical stimulation where the subject started to perceive the same strength as the current EP, was measured. Each measurement was performed 3 times, during which the reproducibility was confirmed, and the mean value of the 3 measurements was used as the measurement value. Through those measurements, Pain Degree (PD) was automatically calculated using the following equation:

PD = 100 × (PEC − CPT)/CPT

### 2.5. Ocular Surface Examinations

The ocular surface examinations in this study were performed in the following order. First, to evaluate the aqueous tear volume over the ocular surface, a video-meniscometer was used to measure the tear meniscus radius (TMR) of the central lower tear meniscus as an index of tear volume [24,25]. In the meniscometry examination, an illuminated target comprising a series of horizontal stripes was projected onto the central lower tear meniscus, and the specular reflex image of the target was then recorded using a digital video recorder. Image analysis software was then used to calculate the TMR via the application of the concave mirror formula.

Next, strict attention was placed on not increasing the subject’s aqueous tear volume; i.e., after 2 drops of saline solution were put on a fluorescein test strip (Ayumi Co., Tokyo, Japan), the strip was vigorously shaken and the central portion of the top of the strip was then gently placed on the central lower lid margin. After several natural blinks, the patient was then verbally instructed to gently close and then briskly open the eye. This verbal instruction was essential in order to clearly determine the starting point (time = 0 s) of the eye opening, as well as to confirm the reproducibility of the FBUP.

To determine the DE subtypes, FBUPs were evaluated simultaneously with the measurement of the fluorescein breakup time (FBUT). As stated above, cases that were not ADDE were excluded from the study. After that, the corneal and conjunctival epithelial damage was scored.

FBUT was measured by 1 evaluator (N.Y.) using a slit-lamp microscope with a cobalt-blue filter for illumination and a blue-free filter for observation [26,27]. An electronic metronome was used for the measurement; i.e., the metronome sound was set to beep every second in order to define the elapsed time from the start until the first appearance of a dark spot in the fluorescein-stained precorneal tear film when the eye was kept open. The FBUT was measured 3 times, and then averaged. When the FBUTs were measured, the FBUPs were evaluated, and those that did not reproduce the same pattern 3 times were excluded. Fluorescein staining of the cornea and conjunctiva was observed using the blue-free filter [26], and then scored based on the severity of the staining. Staining of the cornea was scored in direct reference to the National Eye Institute scoring system [28], in which the cornea was divided into 5 portions and the staining was scored from 0 to 3 at each portion to calculate the total score on scales of 0–15 points. Staining of the conjunctiva was scored in direct reference to the van Bijsterveld scoring system [29], in which the staining was scored independently from 0 to 3 at the nasal and temporal bulbar conjunctiva in order to calculate the total score on scales of 0–6 points. The diagnosis of meibomian gland dysfunction (MGD) was made according to the Japanese diagnostic criteria [30]. Superior limbic keratoconjunctivitis (SLK) and lid-wiper epitheliopathy (LWE) were scored 0–3 according to severity, following the method applied in previous reports [31,32].

In addition, as a test of tear secretion, the Schirmer 1 test was adopted using a standard Schirmer test strip (Ayumi Co., Tokyo, Japan) in which the wetted part of the strip from the top was measured under natural blinking 5 min after the insertion of the strip at the temporal one-third of the lid margin into the conjunctival sac. Finally, an anesthetic eye drop test was performed; i.e., cases in which the EP did not disappear with anesthetic eye drops were defined as positive.

### 2.6. Statistical Analysis

For pre- and post-treatment comparisons, the paired-samples *t*-test was used for parametric and continuous items, and the Wilcoxon signed-rank test was used for non-parametric or intermittent items. For both tests, differences of *p* < 0.05 were considered statistically significant. The correlation coefficient was calculated using the Spearman’s rank correlation coefficient formula, and a *p*-value of <0.05 was considered statistically significant. For analysis of correlations between questionnaires, a *p*-value of <0.05 and a correlation coefficient of ≥0.5 were considered statistically significant. For multivariate analysis, the relative risk was measured using the least squares method, and a *p*-value of <0.05 was considered statistically significant. JMP PRO version 15 (SAS Institute, Inc., Cary, NC, USA) statistics software was used for all statistical analyses.

## 3. Results

### 3.1. Patient Background and Clinical Objective Findings

The background and objective findings of the cases included in this study are shown in Table 1. This study involved 53 eyes of 53 ADDE patients with EP [6 males and 47 females; 26 right eyes and 27 left eyes; mean age: 64.4 ± 13.4 (mean ± SD) years, range: 34 to 86 years]. Of the 53 cases included in the study, 18 eyes of 18 patients were treated with punctal occlusion [1 male and 17 females; 6 right eyes and 12 left eyes; mean age: 67.9 ± 10.2 (mean ± SD) years, range: 49 to 84 years].

After punctal occlusion treatment, corneal and conjunctival epithelial damage other than in the upper cornea were significantly improved compared to before treatment. LWE scores also improved significantly in both the upper and lower eyelid margins. TMR, which reflects tear volume, was also significantly increased, and FBUT was significantly prolonged. After treatment, the Schirmer 1 test was not performed because the treatment precluded accurate measurement.

### 3.2. Pain Subscale of DE-Related Symptoms Evaluated Using DSQ-VAS and SF-MPQ-2 in ADDE

The results of the relationship between DE-related symptoms evaluated using DSQ-VAS and each pain subscale or total pain evaluated using SF-MPQ-2 are shown in Supplementary Table S1. “Eye pain” (VAS) was also significantly correlated with the four pain subscales and total pain (R = 0.63, 0.58, 0.63, 0.59, and 0.75, respectively) (all: *p* < 0.001) using SF-MPQ-2. Moreover, a significant correlation was found between “eye fatigue”/“heavy eyelids” evaluated using DSQ-VAS and continuous pain, affective descriptors, and total pain, respectively, carried out using SF-MPQ-2 (eye fatigue: R = 0.57, 0.66, and 0.60, respectively; heavy eyelids: R = 0.55, 0.54, and 0.55, respectively) (all: *p* < 0.001). Other subjective symptoms that correlated with total pain were “dryness”, “sensitivity to light”, “foreign body sensation”, and “difficulty in opening the eye” (R = 0.56, 0.53, 0.52, and 0.67, respectively) (all: *p* < 0.001).

### 3.3. Pain Degree (PD) Evaluated Using PainVision^®^ and Relationship between PD and Objective Findings

**Figure 1:**
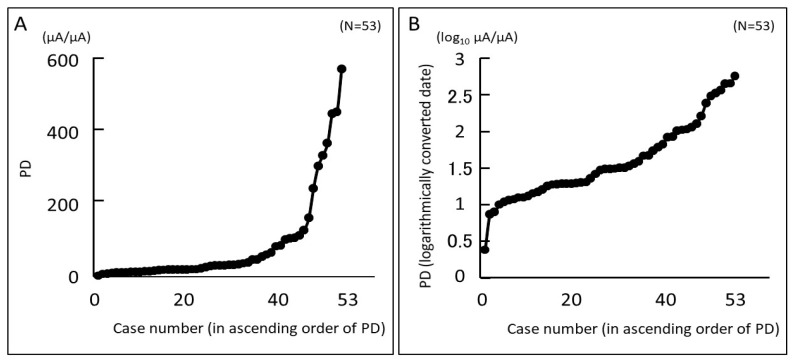
Eye pain (EP) for all subjects evaluated using PainVision^®^. Assessed EP is expressed as a “Pain Degree (PD)” index. (**A**) PD for all subjects is arranged in increasing order from left to right. (**B**) Logarithmic transformation of A. The base of the logarithm is 10. Log-transformed PD increased linearly for all subjects.

Figure 1A shows a graph of the PD of the 53 cases included in this study, sorted from least painful to most painful. PD increased exponentially according to the Weber–Fechner law [33,34]. Figure 1B is a log-transformed graph of Figure 1A. The base of the logarithm was set to 10. Log-transformed PD increased linearly for all subjects. The median PD for the 53 subjects was 30.6 µA/µA (interquartile range, 16.9 to 93.2). When log-transformed with the base of the logarithm as 10, the median PD was 1.49 (interquartile range, 1.23 to 1.97).

The correlation between objective findings and PD is shown in Table 2. The nasal and central corneal staining score and the upper LWE score were significantly correlated with PD (R = 0.33, 0.33, and 0.28, respectively) (all: *p* < 0.05). EP (VAS) assessed using the questionnaire did not correlate significantly with objective findings. According to Spearman’s rank correlation coefficient, central corneal and nasal epithelial disorders were highly correlated (R = 0.69, *p* < 0.0001). Table 3 shows the results of multivariate analysis of objective findings for PD using the least squares method. The central corneal staining score most significantly affected PD.

### 3.4. Changes in PD before and after Punctal Occlusion Treatment

**Figure 2:**
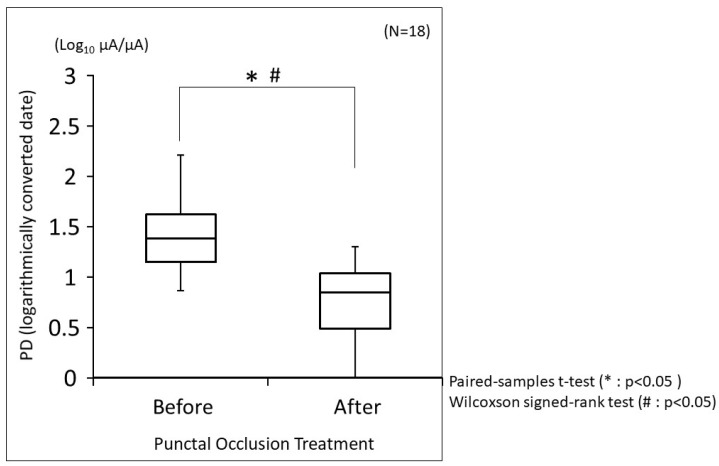
Box-and-whisker plot of minimum to maximum EP evaluated using PainVision^®^. The evaluated EP is expressed by the PD index, and the logarithmic conversion of PD is shown in this figure. The base of the logarithm is 10. The box contains the lower quartile, the median, and the upper quartile before and after punctal occlusion treatment, and the minimum and maximum values are indicated by vertical bars. The paired-samples *t*-test and the Wilcoxon signed-rank test were used for statistical analysis, and a *p*-value of <0.05 was considered statistically significant.

The changes in PD before and after punctal occlusion treatment are shown in Figure 2. The median PD before treatment was 24.8 µA/µA (interquartile range, 14.2 to 42.5). On the other hand, the median PD after treatment was 7.1 µA/µA (interquartile range, 3.1 to 11.3). When log-transformed with the base of the logarithm as 10, the median PD before treatment was 1.39 (interquartile range, 1.15 to 1.62), and the median PD after treatment was 0.85 (interquartile range, 0.49 to 1.29). PD was significantly reduced with treatment (*p* < 0.0001; both the paired-samples *t*-test and the Wilcoxon signed-rank test). When correlations between PD and objective findings were examined for 18 cases at 2 time-points (before and after treatment), for a total of 36 time-points, many objective findings were significantly correlated with PD (Table 4). Table 5 shows the results of multivariate analysis of objective findings for PD using the least squares method. In the punctal occlusion treatment cases, the central corneal staining score and TMR were significantly more influential as factors contributing to PD.

## 4. Discussion

What is clear from the findings in this study is that epithelial damage in the central cornea is the primary cause of EP in ADDE, which is illustrated in the results shown in Table 2 and Table 3. In regard to epithelial damage in the central cornea, it has previously been reported that of the nerve fibers in the whole cornea, they are most dense in the central cornea, and our results support that finding [35]. Moreover, these findings indicate that treating central corneal epithelial damage can contribute to EP relief. However, lower corneal epithelial damage was found to not be such an important finding from the aspect of EP. Therefore, we theorize that a shift in corneal epithelial damage from the central cornea to the lower part of the cornea via some type of treatment (e.g., eye drops) may provide an improved level of EP relief.

Our findings, when comparing before and after punctal occlusion treatment, also supported central corneal epithelial damage as being the most influential factor in EP. As shown in Table 4, many objective findings correlate with PD. This may be due to the fact that punctal occlusion treatment affects many objective findings, especially the objective findings of epithelial damage in the central cornea and TMR, which were found to be significantly associated with PD. TMR reflects aqueous tear volume, and since punctal occlusion treatment increased aqueous tear volume, it may have been significantly correlated with EP. This is a reasonable finding considering that ADDE, as classified by FBUP, represents the most severe form of aqueous tear deficiency. Epithelial damage in the central cornea was most significantly correlated with EP, and may be the factor with the greatest impact on improvement in EP via punctal occlusion treatment. Hence, those findings clearly suggest that punctal occlusion is an effective treatment for severe ADDE classified by BUP to improve the level of EP relief.

In several previous reports on DE, the discrepancy between objective findings and subjective symptoms has been a problem [3,4]. Based on the findings in this study, we consider that there are two reasons for this. First, DE is greatly affected by decreased aqueous tears and decreased wettability of the ocular surface. DWDE causes severe EP without corneal epithelial damage [20,36]. The correlation between EP and central corneal epithelial damage is likely to decrease as DE cases like DWDE with severe EP without corneal epithelial damage are included in the analysis. As evidence to support that speculation, in our previous study on DE with mixed enrollment of DWDE, ADDE, and IEDE cases, we found that many objective findings did not correlate with PD when evaluated in a similar manner [20]. That study included almost equal numbers of ADDE and DWDE cases (ADDE: *n* = 20 cases; DWDE: *n* = 22 cases). Thus, it will be necessary in the future to elucidate objective findings that affect EP in DWDE.

The second reason is the use of questionnaires to assess subjective symptoms. As highlighted by the findings in this study, it was difficult to obtain a correlation between objective findings and EP using the VAS-based questionnaire method of assessing pain (Table 2). The subjective experience of pain is highly variable, even when the underlying tissue damage is identical. According to a study by a Chinese psychology group, the more pain-sensitive group responded to the same level of electrical stimulation with more pain in the questionnaire [37]. Pain sensitivity is influenced by psychological influences (e.g., fear of pain) [37,38,39,40]. Although psychological factors are important for assessing pain, it is important to use an evaluation method in which psychological factors are less likely to intervene when examining the relationship between objective findings and EP. Otherwise, if the subject is the same (e.g., before and after treatment), the psychological factor bias is the same and the questionnaire format can also work somewhat effectively (Supplementary Figure S1). Alternatively, the questionnaire format is also useful when looking for correlations between subjective symptoms that are not related to objective findings (Supplementary Table S1). The questionnaire format can be used to correlate symptoms with EP. In our previous study, we reported that in addition to EP symptoms, “eye fatigue” and “heavy eyelids” were also perceived as pain in various types of DE [20]. In this present study, newly identified subjective symptoms associated with ocular pain in ADDE cases were “dryness”, “sensitivity to light”, “foreign body sensation”, and “difficulty in opening the eye”. In regard to the objective findings associated with PD in ADDE in this study, “sensitivity to light” was considered to be a symptom affected by epithelial damage in the central cornea, and “difficulty opening the eye” was also considered to be due to epithelial damage in the central cornea, which facilitates the blink reflex.

The findings in this study indicate that the classification of DE by FBUP classification is also useful in determining treatment efficacy, as it makes it possible to use epithelial damage in the central cornea as an indicator for improving the symptoms of EP.

It should be noted that this present study did have limitations. First, confocal microscopy was not performed, so it was difficult to evaluate corneal nerve abnormalities such as microneuromas [10,11,41]. Second, the sample size in this report was small, so further studies involving a larger sample size may be needed to confirm the results. Finally, tear film osmolarity was not investigated in this study.

## 5. Conclusions

In conclusion, the findings in this study successfully clarified the relationship between objective findings and EP in ADDE by using the stimulus of electric current to evaluate the subjective symptom of EP, and the objective finding found to be most associated with EP in ADDE is central corneal epithelial damage.

## Figures and Tables

**Table 1:** Demographics and clinical characteristics of the subjects according to the study groups.

		Punctal Occlusion Treatment		
	Total	Before	After	*p* Value
	(*n* = 53)	(*n* = 18)	(*n* = 18)	
Patient Demographics				
Age, yrs, mean (SD)	64.4 (13.4)	67.9 (10.2)		
Female, *n* (%)	47 (88.7%)	17 (94.4%)		
Sjögren’s syndrome, *n* (%)	24 (45.3%)	8 (44.4%)		
Ocular Surface Evaluations				
Corneal staining score (0–15), $	8.3 (3.8)	9.6 (3.1)	3.5 (3.8)	<0.0001
Upper	0.64 (1.0)	0.89 (1.3)	0.56 (0.98)	0.42
Temporal	1.6 (1.2)	1.9 (1.0)	0.44 (0.92)	0.0002
Nasal	1.9 (1.1)	2.3 (0.84)	0.67 (0.97)	<0.0001
Central	2.1 (1.2)	2.6 (0.70)	0.39 (0.85)	<0.0001
Lower	2.1 (0.96)	2.2 (1.0)	0.89 (1.0)	0.0014
Conjunctival staining score (0–6), $	3.8 (2.1)	4.3 (2.0)	1.2 (1.9)	0.0004
Temporal	1.8 (1.1)	2.2 (1.1)	0.56 (1.0)	0.0002
Nasal	2.0 (1.1)	2.1 (1.1)	0.67 (1.0)	0.0007
SLK score (0–3), $	0.32 (0.75)	0.50 (0.92)	0.11 (0.47)	0.09
Upper LWE score (0–3), $	1.1 (1.0)	1.0 (0.97)	0.22 (0.55)	0.0035
Lower LWE score (0–3), $	1.3 (0.96)	1.3 (0.96)	0.5 (0.62)	0.0085
FBUT, seconds $	1.7 (1.6)	0.87 (1.3)	7.4 (3.1)	<0.0001
TMR, mm #	0.15 (0.06)	0.17 (0.07)	0.48 (0.25)	<0.0001
Schirmer 1 test, mm	4.5 (5.9)	3.5 (5.2)		
MGD, *n* (%)	2 (3.8%)	0	0	
Anesthesia test positive, *n* (%)	8 (15.0%)	1 (5.6%)	1 (5.6%)	

#: Student’s *t*-test; $: Wilcoxon test; SLK: superior limbic keratoconjunctivitis; LWE: lid wiper epitheliopathy; FBUT: fluorescein breakup time; TMR: tear meniscus radius; MGD: meibomian gland dysfunction.

**Table 2:** Correlation between ocular surface evaluations and EP.

	Pain Degree (PainVision^®^)		Eye Pain (VAS)	
*n* = 57	R	*p* Value	R	*p* Value
Corneal Staining Score (Total)	0.18	0.19	0.01	0.92
Upper	0.17	0.22	0.01	0.94
Temporal	0.14	0.30	0.02	0.87
Nasal	0.33	0.017	0.18	0.20
Central	0.33	0.014	0.09	0.50
Lower	−0.18	0.19	−0.03	0.82
Conjunctival Staining Score (Total)	0.09	0.54	−0.07	0.60
Temporal	−0.01	0.94	−0.16	0.25
Nasal	0.17	0.23	0.01	0.93
SLK score	−0.12	0.40	−0.16	0.26
Upper LWE score	0.28	0.042	0.08	0.59
Lower LWE score	0.25	0.07	0.06	0.69
FBUT	−0.12	0.41	0.04	0.77
TMR	−0.003	0.98	−0.01	0.92
Schirmer 1 test	−0.009	0.95	0.17	0.23

VAS: visual analog scale; R: Spearman’s rank correlation coefficient; SLK: superior limbic keratoconjunctivitis; LWE: lid wiper epitheliopathy; FBUT: fluorescein breakup time; TMR: tear meniscus radius.

**Table 3:** Multivariate analysis of objective findings for PD using the least squares method.

(*n* = 57)	Multivariate Analysis		Multivariate Analysis 2	
Variables	Logarithmic Value	*p* Value	Logarithmic Value	*p* Value
Age	0.160	0.69	0.210	0.62
Sex (female)	0.055	0.88	0.030	0.93
Central Corneal staining score	1.386	0.04	1.430	0.04
Conjunctival staining score	0.112	0.77		
Upper LWE score	0.940	0.11	0.913	0.12
SLK score	0.131	0.74		
FBUT	0.769	0.17	0.883	0.13
TMR	0.565	0.27	0.626	0.24

LWE: lid wiper epitheliopathy; SLK: superior limbic keratoconjunctivitis; FBUT: fluorescein breakup time; TMR: tear meniscus radius.

**Table 4:** Correlation between ocular surface evaluations and PD before and after punctal occlusion treatment.

	Pain Degree (PainVision^®^)	
*n* = 36	R	*p* Value
Corneal Staining Score (Total)	0.58	0.0002
Upper	0.25	0.14
Temporal	0.61	<0.0001
Nasal	0.60	0.0001
Central	0.69	<0.0001
Lower	0.40	0.02
Conjunctival Staining Score (Total)	0.71	<0.0001
Temporal	0.66	<0.0001
Nasal	0.73	<0.0001
SLK Score	0.43	0.009
Upper LWE Score	0.55	0.0005
Lower LWE Score	0.45	0.006
FBUT	−0.71	<0.0001
TMR	−0.49	0.002
Schirmer 1 test		

R: Spearman’s rank correlation coefficient; SLK: superior limbic keratoconjunctivitis; LWE: lid wiper epitheliopathy; FBUT: fluorescein break up time; TMR: tear meniscus radius.

**Table 5:** Multivariate analysis of objective findings for PD using the least squares method before and after punctal occlusion treatment.

*n* = 36	Multivariate Analysis		Multivariate Analysis 2	
Variables	Logarithmic Value	*p* Value	Logarithmic Value	*p* Value
Age	0.001	0.997	0.139	0.73
Sex (female)	0.404	0.39	0.231	0.59
Corneal staining score				
Upper	0.469	0.34		
Temporal	0.100	0.79		
Nasal	0.379	0.42		
Central	2.020	0.0095	2.060	0.009
Lower	0.444	0.36		
Conjunctival staining score				
Temporal	0.096	0.80		
Nasal	0.798	0.16	0.953	0.11
SLK score	0.009	0.98		
Upper LWE score	0.626	0.24	0.605	0.25
Lower LWE score	0.008	0.98		
FBUT	0.736	0.18	0.441	0.36
TMR	1.466	0.03	1.456	0.04

SLK: superior limbic keratoconjunctivitis; LWE: lid wiper epitheliopathy; FBUT: fluorescein breakup time; TMR: tear meniscus radius.

## Data Availability

The data that support the findings of this study are available from the corresponding author upon reasonable request.

## Supplementary Materials

The following supporting information can be downloaded at: https://www.mdpi.com/article/10.3390/diagnostics14010030/s1, Figure S1: Box-and-whisker plot of minimum to maximum EP evaluated by VAS; Table S1: Correlation between subjective symptoms of dry eye evaluated by VAS and SF-MPQ-2 evaluated by points.
