# Supplementary material for: Eye Pain Caused by Epithelial Damage in the Central Cornea in Aqueous-Deficient Dry Eye
Source: Diagnostics (Basel). 2023 Dec 22;14(1):30. doi: 10.3390/diagnostics14010030 (PMC10802830; doi:10.3390/diagnostics14010030)
Supplement: Supplementary file 1 [file diagnostics-14-00030-s001.zip › Supplementary Table S1.pdf]

Supplementary Table S1. Correlation between subjective symptoms of dry eye evaluated by VAS and SF-MPQ-2 evaluated by points

n=57	SF-MPQ-2									
	Continuous pain		Intermittent pain		Neuropathic pain		Affective descriptors		Total pain	
VAS	R	p	R	p	R	p	R	p	R	p
Dryness	0.37	0.01	0.48	0.0003	0.32	0.02	0.65	<.0001	0.56	<.0001
Blurred vision	0.54	<.0001	0.31	0.03	0.39	0.004	0.33	0.01	0.44	0.001
Sensitivity to light	0.49	0.0002	0.43	0.001	0.44	0.0009	0.44	0.001	0.53	<.0001
Eye fatigue	0.57	<.0001	0.42	0.002	0.36	0.008	0.66	<.0001	0.60	<.0001
Heavy eyelids	0.55	<.0001	0.34	0.01	0.35	0.01	0.54	<.0001	0.55	<.0001
Eye pain	0.63	<.0001	0.58	<.0001	0.63	<.0001	0.59	<.0001	0.75	<.0001
Foreign body sensation	0.41	0.002	0.48	0.0003	0.39	0.004	0.43	0.001	0.52	<.0001
Difficulty in opening the eye	0.53	<.0001	0.55	<.0001	0.49	0.0002	0.58	<.0001	0.67	<.0001
Redness	0.28	0.05	0.18	0.19	0.15	0.28	0.20	0.14	0.25	0.08
Tearing	0.33	0.02	0.22	0.12	0.32	0.02	0.22	0.12	0.33	0.02
Itchiness	0.42	0.002	0.17	0.22	0.19	0.16	0.34	0.01	0.34	0.01
Discharge	0.23	0.09	0.30	0.03	0.14	0.31	0.27	0.05	0.32	0.02

VAS: visual analog scale; SF-MPQ-2: Short-Form McGill Pain Questionnaire 2; R: Spearmant's rank correlation coefficient
Underline is added when the absolute value of R is 0.5 or more.
