# Supplementary figures and images for: Eye Pain Caused by Epithelial Damage in the Central Cornea in Aqueous-Deficient Dry Eye
Source: Diagnostics (Basel). 2023 Dec 22;14(1):30. doi: 10.3390/diagnostics14010030 (PMC10802830; doi:10.3390/diagnostics14010030)

## Slide 1
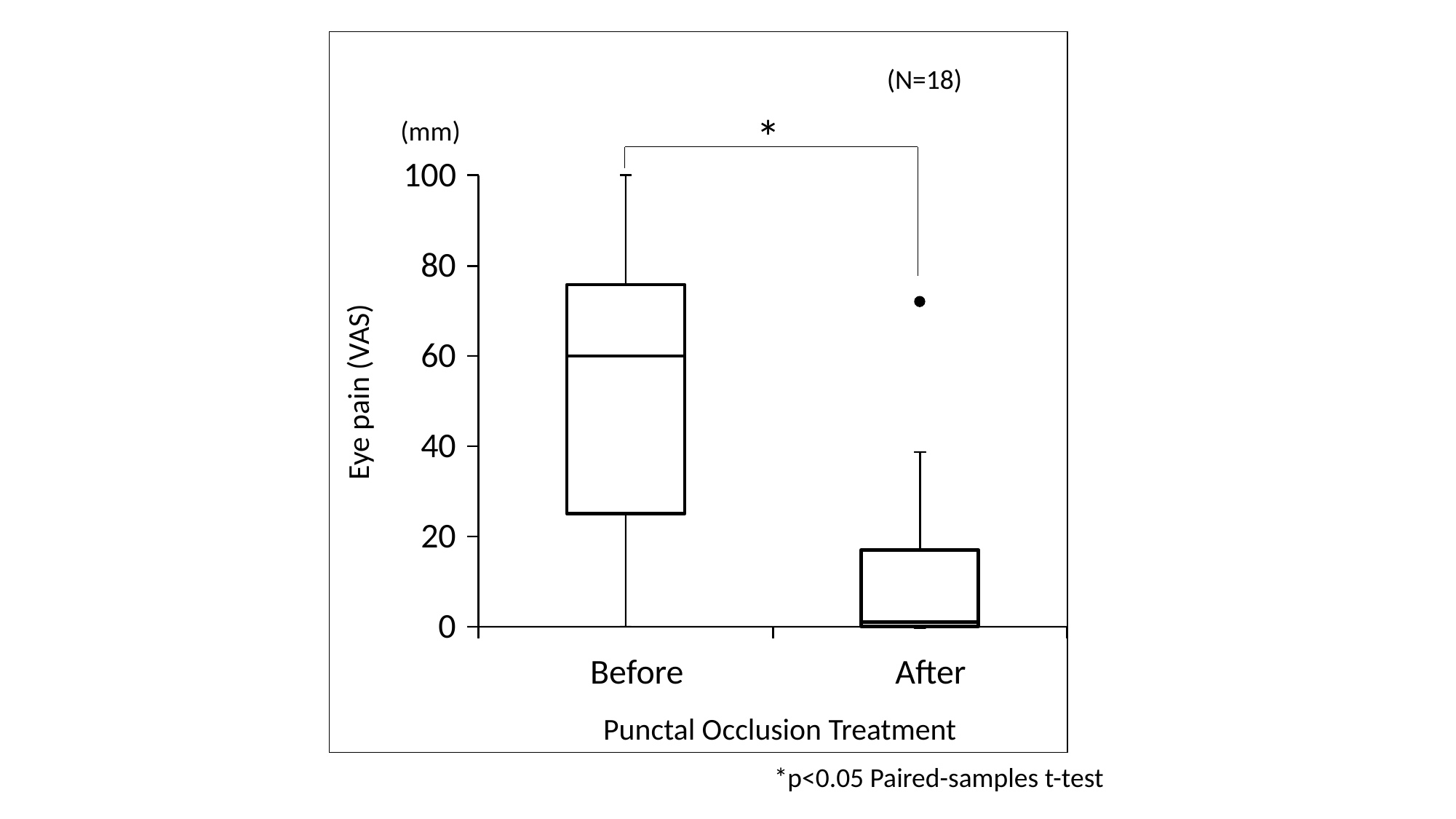

*
(mm)
100
80
60
40
20
0
Before
After
(N=18)
Eye pain (VAS)
 Punctal Occlusion Treatment
*p<0.05 Paired-samples t-test

Supplement: Supplementary file 1 [file diagnostics-14-00030-s001.zip › Supplementary Figure S1.pptx]
